# A Comprehensive Comparative Study for the Authentication of the *Kadsura* Crude Drug

**DOI:** 10.3389/fphar.2018.01576

**Published:** 2019-01-22

**Authors:** Jiushi Liu, Xueping Wei, Xiaoyi Zhang, Yaodong Qi, Bengang Zhang, Haitao Liu, Peigen Xiao

**Affiliations:** ^1^Institute of Medicinal Plant Development, Chinese Academy of Medical Sciences, Peking Union Medical College, Beijing, China; ^2^Key Laboratory of Bioactive Substances and Resources Utilization of Chinese Herbal Medicine (Peking Union Medical College), Ministry of Education, Beijing, China; ^3^Engineering Research Center of Traditional Chinese Medicine Resources, Ministry of Education, Beijing, China

**Keywords:** *Kadsura*, morphology, molecular markers, chemical characterization, identification

## Abstract

The stems and roots of *Kadsura* species have been used as the folk medicine in Traditional Chinese medicine (TCM) and have good traditional efficacy and medicinal application with a long history. Among these species, *K. coccinea*, *K. heteroclita* and *K. longipedunculata* are the most widely distributed species in the regions of south and southwest China. Owing to their similar appearance, the crude drugs are often confusedly used by some folk doctors, even some pharmaceutical factories. To discriminate the crude drugs, haplotype analysis based on cpDNA markers and ITS was firstly employed in this study. Generic delimitation, interspecific interrelationships, and the identification of medicinal materials between *K. longipedunculata* and *K. heteroclita* remained unresolved by the existing molecular fragments. The original plant could be identified through the morphological character of flower, fruit and leaf. However, in most situation collectors have no chance to find out these characters due to lack of reproductive organs, and have no experience with the minor difference and transitional variation of leaf morphology. The chemical characterization show that the chemometric of chemical composition owned higher resolution to discriminate three herbs of *Kadsura* species. In conclusion, this integrative approach involving molecular phylogeny, morphology and chemical characterization could be applied for authentication of the *Kadusra*. Our study suggests the use of this comprehensive approach for accurate characterization of this closely related taxa as well as identifying the source plant and confused herbs of TCM.

## Introduction

Traditional Chinese medicine (TCM) is widely accepted in the health care system, and has made a significant contribution to prevention and treatment of human diseases. This extensive use warrants safety measures and so TCM drug safety monitoring and quality control are becoming increasingly important tasks to guarantee the safety and efficacy of TCM treatments (Chen et al., 1999). However, the use of substitute products and confused materials still aggravate the chaotic situation in clinical application. It is important to find a reliable way for distinguishing them from each other (Li et al., 2015).

*Kadsura* belongs to the economically and medicinally important family Schisandraceae and eight species mainly distributed in the southwest and southeast in China (Saunders, 1998; Wu et al., 2008). In China, the stems and roots of genus *Kadsura* are commonly used as folk medicines and 5 species of genus *Kadsura* are documented in the official Pharmacopoeia and folk record (GuangXi Zhuang Autonomous Region Health Department, 1992; Guangdong Food and Drug Administration, 2004; FuJian Food and Drug Administration, 2006; Chinese Pharmacopeia Commission, 2015). The stems of *K. heteroclita*, *K. longipedunculata* and *K. coccinea* are the most widely used in south China which have good traditional efficacy and medicinal application with a long history, and often used confusedly in clinical application. Numerous phytochemical and pharmacological studies have been carried out and focus on its health benefits (Liu et al., 2014). There are differences in clinical efficacy between the three crude drugs. *K. heteroclita* was used for the treatment of rheumatic arthralgia. *K. longipedunculata* was used for the treatment of irregular menstruation. *K. coccinea* was used for the treatment of gastric and duodenal ulcer. Owing to their similar appearance, these crude drugs are often confusedly used by some folk doctors, even some pharmaceutical factories. There is an urgent need to find a reliable, accurate way for distinguishing three *Kadsura* crude drugs (Liu et al., 2012).

In order to identify this kind of medicine herbs by molecular sequences, Zhou et al. chose psbA-trnH for distinguishing eight species. Although they found a stabilized single nucleotide polymorphism (SNP), SNPs as potential tool to distinguish *Kadsrua* crude drugs could not be further analyzed because of poor samples of *K. heteroclite* (Zhou et al., 2016). Combination of ITS + psbA-trnH + matK + rbcL as the most ideal DNA barcode for discriminating the medicinal plants of Schisandra and *Kadsura*, nonetheless, degree of species resolution was lower among the closely related species, and exposed *K. heteroclita* and *K. longipedunculata* could not be discriminated by four commonly used DNA barcodes (Zhang et al., 2015). Molecular identification of TCM is objective, more accurate, and easier to perform than traditional identification methods, and has successfully been applied to identify medicinal plants (Kress et al., 2005; Chen et al., 2010; de Boer et al., 2015). However, previous studies of DNA barcoding have not effectively resolved the problem of identifying three crude drugs of the *Kadsura*. In this study, we try to identify three crude drugs by haplotype analysis based on cpDNA and ITS markers.

The high selectivity and sensitivity of UPLC-QTOF/MS has been successfully applied to the metabolite analysis and identification of complex compounds in herbal materials (Song et al., 2013; Yao et al., 2013; Cubero-Leon et al., 2014). There are few researches in investigated chemical profiles of *Kadsura* species for the safety and efficacy, and the comparative analysis on chemical composition of these *Kadsura* herbs is needed. Bioactivity-based characteristics are good quality indicators too, as they are pharmacologically relevant (Liu et al., 2017). Analysis on main chemical components disparity in three medicinal materials could guarantee the clinical uses the medicine the rationality, the security and the validity.

Adequately considering samples representativeness and experiments economy, we therefore started multi-populations survey in twenty populations covering five provinces including Hunan, Guangxi, Guizhou, Chongqing and Sichuan provinces and in reproductive stage at summer and autumn from June 2016 to December 2017. The overall aim of this study was to explore the usefulness of an authentication approach to three crude drugs of the *Kadsura* complex using cpDNA and ITS markers, morphology, and UPLC-QTOF/MS chemical profiling. We want to compare the genetic polymorphism of haplotypes and analyze their population difference for the taxa in the *Kadsura* species, and distinguish chemotypes of the species complex by comparing their UPLC-QTOF/MS chemical profiles using chemometric data analysis.

## Materials and Methods

### Plant Materials

There are morphological differences between the three species to discriminate them during flower or fruit stages of life cycles. For the sake of sampling accuracy, we therefore started multi-populations survey in reproductive stage at summer and autumn from June 2016 to December 2017. The *Kadsura* samples were collected in the main areas in China: Hunan, Guangxi, Guizhou, Chongqing and Sichuan provinces. In total 52 samples of *K. coccinea*, *K. heteroclita* and *K. longipedunculata* were collected directly from wild region. The 52 leaves dried using silica gel for DNA extraction and stored at 4°C until use. And 18 the stems dried in the shade for UPLC-MS analysis (Table 1).

**Table 1:** Samples of *K. longipedunculata*, *K. coccinea*, and *K. heteroclita*.

No.	Species	Voucher number	Sources
S1	*K. longipedunculata*	2015082801	Nanchuan, Chongqin
S2	*K. longipedunculata*	2015082903	Emei, Sichuan
S3	*K. longipedunculata*	2015090604	Guiyang, Guizhou
S4	*K. longipedunculata*	2015090801	Leishan, Guizhou
S5	*K. longipedunculata*	2015090804	Baojing, Hunan
S6	*K. longipedunculata*	2015090807	Xingan, Guangxi
S7	*K. coccinea*	2015082901	Jinxiu, Guangxi
S8	*K. coccinea*	2015083101	Jinxiu, Guangxi
S9	*K. coccinea*	2015090502	Emei, Sichuan
S10	*K. coccinea*	2015091601	Guiyang, Guizhou
S11	*K. coccinea*	2015092304	Huaihua, Hunan
S12	*K. coccinea*	2015092704	Xingan, Guangxi
S13	*K. heteroclita*	2015091201	Jianhe, Guizhou
S14	*K. heteroclita*	2015091803	Baojing, Hunan
S15	*K. heteroclita*	2015091804	Jinxiu, Guangxi
S16	*K. heteroclita*	2015092104	Jinxiu, Guangxi
S17	*K. heteroclita*	2015092105	Nanchuan, Chongqin
S18	*K. heteroclita*	2015091201	Emei, Sichuan


### DNA Extraction, PCR Amplification and Sequencing

Total genomic DNA was extracted from silica gel-dried leaves by using the Plant Genomic DNA Kit (Tiangen Biotech, Beijing, China) following the manufacturer’s instructions. Three cpDNA gene markers, matK,rbcL,psbA-trnH and one nrDNA ITS, were separately amplified for each individual by using the primers and protocol of Guo et al. (2015). Sanger sequence reactions were carried out using the DYEnamic ETDye Terminator Cycle Sequencing Kit (Amersham Pharmacia Biotech) and sequenced on ABI 3730XL genetic analyzer (Applied Biosystems, CA, United States).

### Network Analysis of Haplotypes

The DNA sequences were aligned using the program Clustal X v.1.83 (Thompson et al., 1997) and manually adjusted in BioEdit v. 7.0.9 (Hall, 1999). Voucher and GenBank accession numbers were listed in the Supplementary Table S1. A network of the cpDNA haplotypes (chlorotypes) was constructed using NETWORK 5.0.0.1 (Bandelt et al., 1999), with a default parsimony connection limit of 95% and each insertion/deletion (indel) treated as a single mutation event.

### Sample Preparation and UPLC-QTOF/MS Conditions

HPLC-grade acetonitrile (Merck KGaA, Darmstadt, Germany) and formic acid (Fisher Scientific, NH, United States) were utilized for UPLC analysis. Pure water (18.2 MΩ) for UPLC analysis was obtained from a Milli-Q system (Millipore, MA, United States). All other chemicals were of analytical grade.

*Kadsura* samples (0.5000 g, 65-mesh) were accurately weighed and extracted with 25 mL methanol by ultrasonication (35 kHz) for 30 min. After centrifugation at 10,000 ×*g* for 10 min, the supernatant was stored at 4°C and filtered through 0.22 μm membrane prior to injection into the UPLC system.

A Thermo Scientific^TM^ Dionex^TM^ UltiMate^TM^ 3000 Rapid Separation LC (RSLC) system performed UHPLC separations using the gradient conditions as follows. Mobile phase A was water and mobile phase B was acetonitrile; both A and B contained 0.1% formic acid. The conditions were optimized as follows: 0–3 min, 2–20% B; 3–4.5 min, 20–75% B; 4.5–6.5 min, 75–100% B; 6.5–15 min, 100% B; 15–15.5 min, 100–5% B; 15.5–17 min, 5% B. The column was a HSS T3 column (2.1 mm × 100 mm, 1.7 μm, waters) operated at 45°C. The flow rate was 300 μL/min and the injection volume was 2 μL.

A Thermo Scientific^TM^ Q Exactive^TM^ hybrid quadrupole Orbitrap mass spectrometer equipped with a HESI-II probe was employed. The HESI-II spray voltages were 3.7 kV for positive mode, the heated capillary temperature was 320°C, the sheath gas pressure was 30 psi, the auxiliary gas setting was 10 psi, and the heated vaporizer temperature was 300°C. Both the sheath gas and the auxiliary gas were nitrogen. The collision gas was argon at a pressure of 1.5 mTorr. The parameters of the full mass scan were as follows: a resolution of 70,000, an auto gain control target under 1 × 10^6^, a maximum isolation time of 50 ms, and an m/z range 150–1500. The calibration was customized for the analysis of Q Exactive to keep the mass tolerance of 5 ppm. The LC-MS system was controlled using Xcalibur 2.2 SP1.48 software (Thermo Fisher Scientific), and data were collected and processed with the same software.

### UPLC-QTOF/MS Data Analysis

UPLC-QTOF/MS data for *Kadsura* samples were analyzed to identify potential discriminant variables. Peak finding, alignment and filtering of ES raw data were carried out using Xcalibur 2.2 SP1.48 software (Thermo Fisher Scientific). The parameters used were as follows: retention time (tR) of 0.5–10.5 min, mass of 150–800 Da, retention time tolerance of 0.05 min, and mass tolerance of 0.02 Da. Three replicate samples collected from each geographic location were used (*n* = 3). A total of three, 114 variables were used to create the model. The resulting data was analyzed by heatmap analysis with MetaboAnalyst, which is a web-based tool for visualization of chemometrics (Deng et al., 2014). And principal component analysis (PCA) and partial least squares discriminant analysis (PLS-DA) were applied to discriminate three *Kadsura* species by the EZinfo 2.0 software (Masssart et al., 1998; Xia et al., 2012).

## Results

### Morphology

**FIGURE 1:**
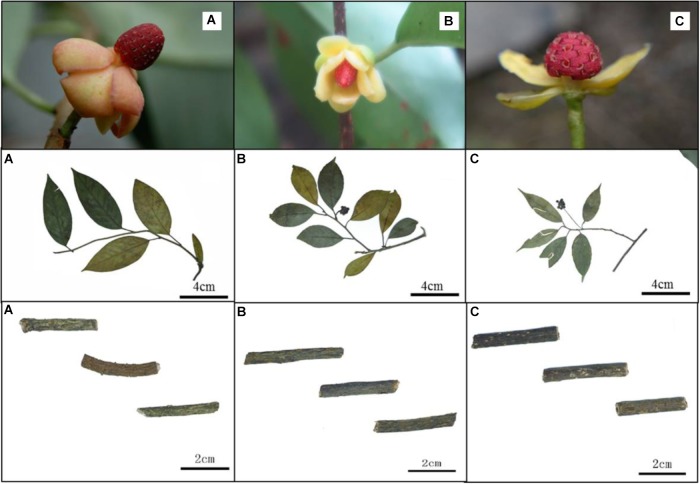
Morphology of *K.* *coccinea* **(A)**, *K.* *heteroclita* **(B)** and *K.* *longipedunculata* **(C)**.

The variation of morphological traits in the *Kadsura* is relatively complex and the *Kadsura* in relationship was near with each other. There are still some morphological differences among three species. We observed main morphology characters (male flower, fruit shape, stem and leaf listed in Table 2) of *K. coccinea*, *K. heteroclita* and *K. longipedunculata* by specimens and natural populations, and established morphological basis to identify three *Kadsura* species. The morphology of male flower, fruit shape, stem and leaf are an important basis of identifying *Kadsura* (Figure 1). In most situations, collectors have no chance to find out these characters because of comparatively short flower and fruit time or lack of these organs in some habits and young individuals. Minor difference and transitional variation of leaf morphology make it difficult for those inexperienced collectors to identify. It is important to find a reliable, accurate way for distinguishing three crude drugs.

**Table 2:** The main morphology characters of *K. coccinea*, *K. heteroclita* and *K. longipedunculata.*

	*K. coccinea*	*K. heteroclita*	*K. longipedunculata*
Petiole	0.9–3 (–4.1) cm	0.7–2.9 cm	0.6–1.7 (–3) cm
Leaf blade	elliptic to rarely ovate; papery to leathery; margin entire or rarely denticulate; apex acute; shortly acuminate, or rarely obtuse	ovate-elliptic to elliptic; papery to subleathery; margin entire or denticulate; apex acute to acuminate	elliptic to rarely ovate-elliptic or obovate-elliptic; papery to leathery; secondary veins 4–8 on each side of midvein; margin subentire, denticulate, serrulate, or serrate; apex shortly to long acuminate
Staminate	stamens 10–50; staminodes generally present at apex of torus	stamens 40–74; staminodes absent	stamens 26–54; staminodes absent
Fruit	6–10 cm; apocarps red to purplish red	2.5–4 cm; apocarps red	1–3.5 cm; apocarps red, purple, or rarely black
Stem	black or brown; lenticel, no suberinlamellae	brown;older stems phellem layer thickness, longitudinal split	slender


### Haplotypes Network

**FIGURE 2:**
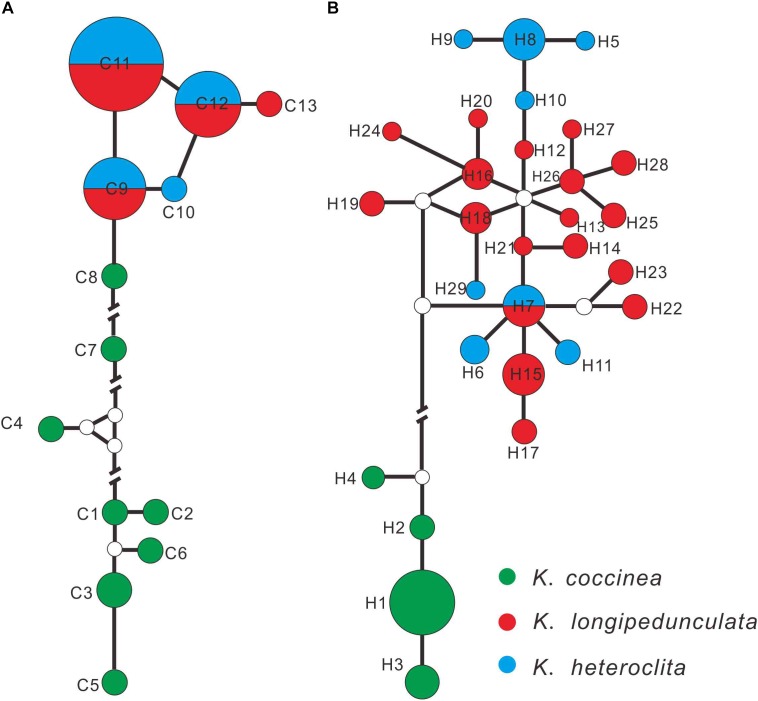
Networks of the cpDNA **(A)** and ITS **(B)** haplotypes constructed by using NETWORK 5.0.0.1. The sizes of the circles in the network are proportional to the observed frequencies of the haplotypes.

The alignment of the combined three cpDNA fragments were designated 13 haplotypes (C1–C13) including 52 variable characters, and ITS were designated 29 haplotypes (H1–H29) with 49 variable characters. *K. coccinea* occupied eight private chloroplastic haplotypes (Figure 2A; C1–C8), while *K. longipedunculata* and *K. heteroclita* shared the three main haplotypes (C9, C11, and C12), two rare haplotypes C10 and C13 were fixed by *K. heteroclita* and *K. longipedunculata*, respectively. In the ITS haplotypes network (Figure 2B), *K. coccinea* occupied four private haplotypes which were quite different from the others (H1–H4). 17 haplotypes were fixed in *K. longipedunculata*, and seven haplotypes in *K. heteroclita*. Only one H17 was shared by *K. longipedunculata* and *K. heteroclita*. H17 was one of the main haplotypes of ITS and it was the center of the “star-like” haplotypes network of *K. longipedunculata* and *K. heteroclita*. It was obviously to see that both cpDNA markers and ITS can distinguish *K. coccinea* from *K. longipedunculata* and *K. heteroclita* clearly. However, the phylogenetic relationship between *K. longipedunculata* and *K. heteroclita* are quite closely related, their haplotypes are star-like and they shared the main haploptypes in both cpDNA markers and ITS. (Supplementary Table S2)

### UPLC-QTOF/MS Analysis

Twenty compounds were tentatively identified by elucidating the retention time (min), parentions [M+H]^+^, MS/MS fragmentation pattern and calculated molecular formula of each peak, and by matching above data with those reported previously (Table 3). For example, schisandrin, in the low energy spectrum, the protonated adduct ion [M+H]^+^ at m/z 433.1866. Further confirmation of schisandrin was provided by the high-energy function. At m/z 415.2115 was detected the fragment identified as [M-H_2_O+H]^+^ and at m/z 384.1932 we assigned a fragment due to the further loss of methoxy group corresponding to [M-H_2_O-OCH_3_+H]^+^. It was identified to schisandrin based on the parent and characteristic fragmentions information.

**Table 3:** Tentatively identified compounds from *K. longipedunculata*, *K. heteroclita*, and *K. coccinea*.

No.	*t*_R_(min)	Mean measured mass [M+H]^+^ m/z	Theoretical exact mass [M+H]^+^ m/z	Error (ppm)	Fragments m/z	Formula	Identification	Reference
1	4.25	445.1861	445.1862	–0.22	355.1545, 337.1440, 323.1283	C_24_H_28_O_8_	kadsumarin A	Kuo et al., 1999
2	4.34	419.2073	419.2070	0.72	269.1542, 234.1409, 206.1096	C_23_H_30_O_7_	gomisin H	Li et al., 1985
3	4.35	415.1390	415.1393	–0.72	291.1385, 273.1279, 247.1123	C_22_H_22_O_8_	kadsurindutin H	Ma et al., 2009
4	4.37	433.1866	433.1862	0.92	313.1440, 279.1385, 253.1229	C_23_H_28_O_8_	kadangustin L	Gao et al., 2012
5	4.41	433.2219	433.2226	–1.62	415.2044, 384.1856, 369.1677	C_24_H_32_O_7_	schisandrin	Sun et al., 2011
6	4.45	387.1809	387.1808	0.26	297.1491, 279.1385	C_22_H_26_O_6_	gomisin M1	Han et al., 1992
7	4.59	625.2071	625.2074	–0.48	317.0814, 291.0657	C_36_H_32_O_10_	angustifolin A	Chen et al., 1998
8	4.76	505.1870	505.1862	1.58	321.1127, 295.0970	C_29_H_28_O_8_	interiotherin A	Chen et al., 1998
9	4.87	431.2070	431.2070	0.00	251.1436, 225.1279	C_24_H_30_O_7_	schisanlignone A	Liu et al., 1991
10	5.15	415.1758	415.1757	0.24	295.1334, 269.1178	C_23_H_26_O_7_	kadsulignan L	Liu and Li, 1995
11	5.18	389.1974	389.1964	2.6	357.1719	C_22_H_28_O_6_	gomisin J	Chen et al., 2006
12	5.28	459.2017	459.2019	–0.44	339.1596, 321.1491, 308.1412	C_25_H_30_O_8_	ananolignan A	Yang et al., 2011
13	5.42	581.2385	581.2387	–0.34	458.1941, 308.1412, 290.1307, 277.1229	C_32_H_36_O_10_	kadangustin E	Gao et al., 2008
14	5.61	417.1556	417.1549	1.68	327.1232, 291.1021	C_22_H_24_O_8_	kadoblongifolin B	Liu et al., 2009
15	5.73	637.2645	637.2649	–0.63	517.2226, 394.1780, 376.1675, 350.1518, 336.1362	C_35_H_40_O_11_	schisantherin J	Liu and Pan, 1991
16	6.15	483.2022	483.2019	0.62	423.1808	C_27_H_30_O_8_	heteroclitin D	Chen et al., 1992
17	6.21	537.2115	537.2125	–1.86	415.1545, 316.1099	C_30_H_32_O_9_	gomisin C	Chen et al., 1992
18	7.57	499.2329	499.2332	–0.60	379.1909	C_28_H_34_O_8_	heteroclitin B	Chen et al., 1992
19	7.65	485.2181	485.2175	1.24	425.1964	C_27_H_32_O_8_	kadsulignan J	Liu et al., 2009
20	7.95	607.2181	607.2179	0.33	517.1862, 394.1933, 360.1878, 346.1722	C_33_H_34_O_11_	kadsuphilol L	Shen et al., 2009


**FIGURE 3:**
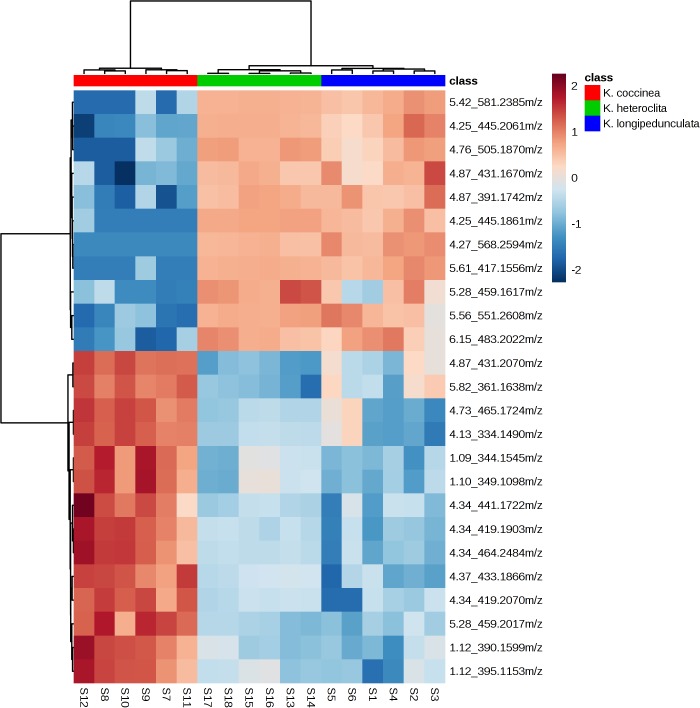
The heatmap presenting the distribution of constituents in *K*. *longipedunculata*, *K. coccinea* and *K. heteroclita.*

As depicted in Figure 3, we can observe that: (a) the chemical components of three *Kadsura* species were very different by the heatmaps, and the components of *K. longipedunculata* were close to *K. heteroclita*. (b) Among all the identified compounds, kadangustin L, gomisin H, and ananolignan A have a relatively high concentration in *K. coccinea*, while showing low levels in the *K. heteroclita* and *K. longipedunculata*. While kadangustin E, kadsumarin A, interiotherin A and kadoblongifolin B are present mainly in the *K. longipedunculata*, followed by the *K. heteroclita* and limited concentratins in the *K. coccinea*. Previous studies have suggested that these compounds are found in high concentrations in *K. heteroclita*, which is supported by our results (Liu et al., 2014).

**FIGURE 4:**
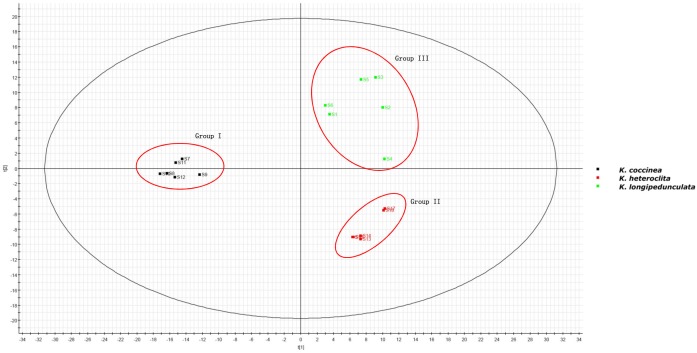
The PCA of *K. longipedunculata, K. coccinea and K. heteroclita.*

The two-component PCA model cumulatively accounted for 46.04% of the variation (PC1, 36.43%; PC2, 9.61%). The PCA score plot shows that these three species were obtained the very good separation. Group I was formed by *K. coccinea*, Group II consisted of *K. heteroclita* and Group III was formed by *K. longipedunculata* (Figure 4).

**FIGURE 5:**
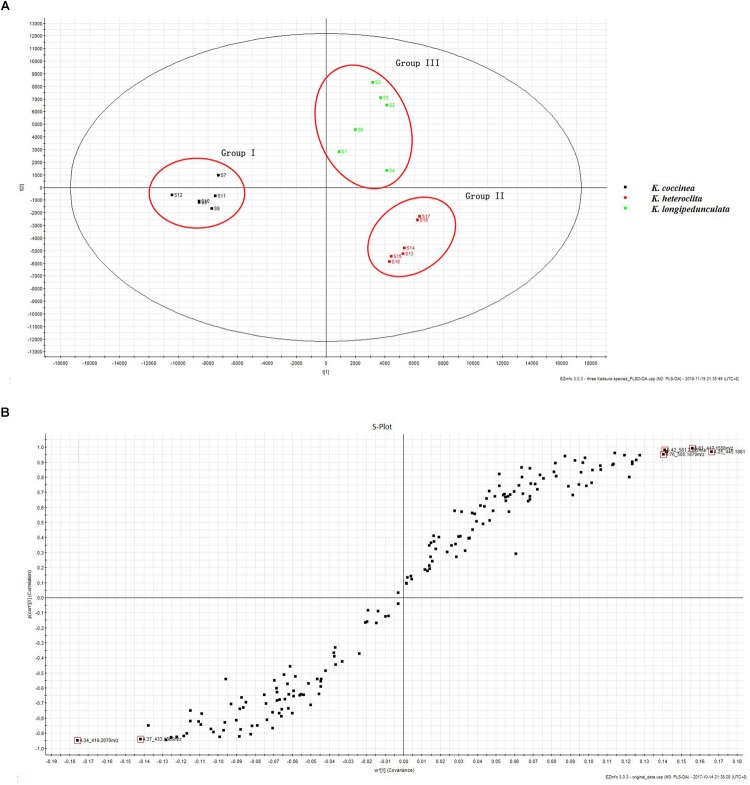
The PLS-DA **(A)** and S-Plot **(B)** of *K. longipedunculata*, *K. coccinea* and *K. heteroclita*.

**FIGURE 6:**
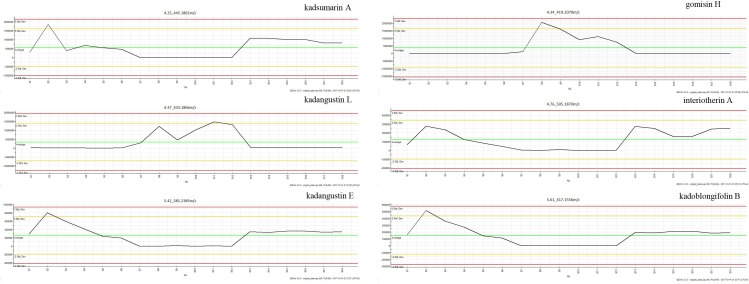
The ion intensities of markers.

The PLS–DA model performed well in classifying the three species of *Kadsura*, and group I was far away from the group II and III (Figure 5A). A total of six credible and significant markers were determined to facilitate discrimination of these groups by the S-plot of PLS-DA. The identities of six potential markers were tentatively assigned (Figure 5B). The components correlated with these six ions were tentatively identified as isomers of kadsumarin A, gomisin H, kadangustin L, interiotherin A, kadangustin E, and kadoblongifolin B. The marker compounds could be used to distinguish the three plant species, as the ion intensities of kadsumarin A, kadoblongifolin B, and interiotherin A in *K. heteroclita* and *K. longipedunculata* was higher than in *K. coccinea*. Marker gomisin H and kadangustin L could be detected in *K. coccinea*, which was higher than in the other two species (Figure 6 and Supplementary Data Sheet S1).

## Discussion

The three *Kadsura* species distribute widely in tropical or subtropical evergreen forests of south of Yangtze River in China. In fact, there are morphological differences between the three species to discriminate them during flower or fruit stages of life cycles. For the sake of sampling accuracy, we therefore started multi-populations survey in reproductive stage at summer and autumn from 2016 June to December 2017. Adequately considering samples representativeness and experiments economy, we chose following survey and sampling strategy. 80 individuals were observed in twenty populations covering five provinces including Hunan, Guangxi, Guizhou, Chongqing and Sichuan provinces, while 45 leaves samples were collected for DNA barcoding experiment and 18 stems samples were used for metabolites analysis, in which samples of each species included ten individuals (DNA test) and six individuals (Chemical test) from different populations.

### Morphology

As mentioned above, we usually discriminate the three species by the shape of staminate flower torus, the size and shape of fruits, the length of fruit stalk and leaf shape. For example, the shape of staminate flower torus of *K.* *coccinea* is conical, *K.* *heteroclite* is elliptical and *K.* *longipedunculata* is spherical. The size of fruit is *K.* *coccinea* (6–10 cm) > *K.* *heteroclita* (2.5–4 cm) > *K.* *longipedunculata* (1–3.5 cm). These identifying characteristics also were record in FRPS and FOC (Academiae Sinicae Edita, 2004; Flora of China Edita, 2013). When we surveyed in wild populations, these morphological characters were very valuable to discriminate them. In spite of obvious differences between reproductive organs, in most situation collectors have no chance to find out these characters due to comparatively short flower and fruit time or lack of these organs in some habits and young individuals. In addition, leaf morphology may be observed through whole growth period, minor difference and transitional variation between species make difficult for those inexperienced collectors. Consequently this leads to collection uncertainty for the three crude drugs, and confusedly mixed application often occurs in present research and clinic use. Nowadays a popular solution is to extract DNA fragments from dried materials, and then conducts DNA barcoding or SNPs analysis (Kress et al., 2005; Chen et al., 2010).

### DNA Sequence

In our study, haplotype analysis based on cpDNA and ITS markers can distinguish clearly *K.* *coccinea* from *K.* *longipedunculata* and *K.* *heteroclita*, but can’t distinguish *K.* *longipedunculata* and *K.* *heteroclite*. Haplotype analysis is suitable for the study of closely related species and genetic diversity of intraspecific species by molecular biology methods. However, it does not show any advantage to delimit the boundary between *K. longipendunculata* and *K. heteroclita*. Haplotypes of *K. longipedunculata* and *K. heteroclita* shared the main haploptypes in both cpDNA markers and ITS. MatK,rbcL,psbA-trnH and ITS are the four suggested DNA barcode in plant (CBOL Plant Working Group, 2009). The cpDNA is characterized by its evolutionary conservatism, matrilineal inheritance, and lack of recombination (Wolfe et al., 1987). However, the complicated relationship such as the potential hybridization, reticulate evolution and gene introgression may further intersify the difficulty of species identification in closely related species of the *Kadsura*. The existing DNA barcode have not effectively resolved the problem of identifying *K. longipedunculata* and *K. heteroclita*.

### Chemical Characteristics

Three herbs differ from their metabolite profiles including lignan based chemometric analysis. Heatmap analysis, PCA analysis and PLS-DA showed the chemical constituents of three kinds of medicinal materials differed significantly. We summarized the chemical constituents of *Kadsura* and found that a lot of spirobenzofuranoid dibenzocyclooctadiene compounds have been found in *K. heteroclita*. Tetrahydrofura compounds have been found in *K. longipedunculata*. 18 (13→12)-abeo-lanostane and nortriterpenoid compounds have been found only in *K. coccinea* (Liu et al., 2014). The different chemical constituents can influence the curative effect and security. The chemometrics analysis can make up for the shortage of molecular identification and has successfully been applied to identify the three *Kadsura* crude drug.

In this study, the DNA sequence analyzes, the recheck of morphology and chemical characteristics applied to identify the three *Kadsura* crude drug. The identification of medicinal materials between *K. longipedunculata* and *K. heteroclita* remained unresolved by the existing molecular fragments. The chemical characterization shows that the chemometric of chemical composition owned higher resolution to discriminate three crude drugs of the *Kadsura* and helpful to differentiate the source of samples and judge the consistency of three *Kadsura* species which make up for the shortage of molecular identification. This paper conducts a comprehensive analysis on three *Kadsura* crude drugs and provides a new research route for the confused herbs by molecular phylogeny, morphology and chemical composition.

## Author Contributions

JL involved field survey, performed operation of the whole experiments, and wrote the manuscript. XZ and XW assisted with JL in the experiments. YQ and HL responsible for provided the technical guidance and designed the experiments. BZ and PX improved the manuscript.

## Conflict of Interest Statement

The authors declare that the research was conducted in the absence of any commercial or financial relationships that could be construed as a potential conflict of interest.
