# Supplementary material for: A Comprehensive Comparative Study for the Authentication of the Kadsura Crude Drug
Source: Front Pharmacol. 2019 Jan 22;9:1576. doi: 10.3389/fphar.2018.01576 (PMC6357937; doi:10.3389/fphar.2018.01576)
Supplement: Supplementary file 1 [file Table_1.DOCX]

**Supplementary Table S1.** Taxa, voucher and GenBank accession numbers for the sequences used in this study

| Species | Voucher number | GenBank accession numbers | | | |
| --- | --- | --- | --- | --- | --- |
|  |  | rbcL | psbA-trnH | matK | ITS |
| *Kadsura coccinea* | 2015072001 | KY884912 | KY884847 | KY884781 | KY884717 |
| *Kadsura coccinea* | 2015071901 | KY884913 | KY884848 | KY884782 | KY884718 |
| *Kadsura coccinea* | 2015082801 | KY884914 | KY884849 | KY884783 | KY884719 |
| *Kadsura coccinea* | 2015082802 | KY884915 | KY884850 | KY884784 | KY884720 |
| *Kadsura coccinea* | 2015083001 | KY884916 | KY884851 | KY884785 | KY884721 |
| *Kadsura coccinea* | 2015091502 | KY884917 | KY884852 | KY884786 | KY884722 |
| *Kadsura coccinea* | 2015091601 | KY884918 | KY884853 | KY884787 | KY884723 |
| *Kadsura coccinea* | 2015092104 | KY884919 | KY884854 | KY884789 | KY884724 |
| *Kadsura coccinea* | 2015083101 | KY884920 | KY884855 | KY884790 | KY884725 |
| *Kadsura coccinea* | 2015090502 | KY884921 | KY884856 | KY884791 | KY884726 |
| *Kadsura coccinea* | 2015091601 | KY884922 | KY884857 | KY884792 | KY884727 |
| *Kadsura coccinea* | 2015092304 | KY884923 | KY884858 | KY884793 | KY884728 |
| *Kadsura heteroclita* | 2015090202 | KY884925 | KY884860 | KY884813 | KY884730 |
| *Kadsura heteroclita* | 2015090203 | KY884926 | KY884861 | KY884814 | KY884731 |
| *Kadsura heteroclita* | 2015090204A | KY884927 | KY884862 | KY884815 | KY884732 |
| *Kadsura heteroclita* | 2015090204B | KY884928 | KY884863 | KY884816 | KY884733 |
| *Kadsura heteroclita* | 2015090208 | KY884929 | KY884864 | KY884817 | KY884734 |
| *Kadsura heteroclita* | 2015091201 | KY884930 | KY884865 | KY884794 | KY884735 |
| *Kadsura heteroclita* | 2015091803 | KY884931 | KY884866 | KY884795 | KY884736 |
| *Kadsura heteroclita* | 2015091804 | KY884932 | KY884867 | KY884796 | KY884737 |
| *Kadsura heteroclita* | 2015092104 | KY884933 | KY884868 | KY884797 | KY884738 |
| *Kadsura heteroclita* | 2015092105 | KY884934 | KY884869 | KY884798 | KY884739 |
| *Kadsura heteroclita* | 2015092106 | KY884935 | KY884870 | KY884799 | KY884740 |
| *Kadsura longipedunculata* | 2015082801 | KY884946 | KY884881 | KY884810 | KY884752 |
| *Kadsura longipedunculata* | 2015082903 | KY884947 | KY884882 | KY884811 | KY884753 |
| *Kadsura longipedunculata* | 2015083102 | KY884948 | KY884883 | KY884812 | KY884754 |
| *Kadsura longipedunculata* | 2015090602 | KY884949 | KY884884 | KY884818 | KY884755 |
| *Kadsura longipedunculata* | 2015090604 | KY884950 | KY884885 | KY884819 | KY884756 |
| *Kadsura longipedunculata* | 2015090801 | KY884951 | KY884886 | KY884820 | KY884757 |
| *Kadsura longipedunculata* | 2015090802 | KY884952 | KY884887 | KY884821 | KY884758 |
| *Kadsura longipedunculata* | 2015090803 | KY884953 | KY884888 | KY884822 | KY884759 |
| *Kadsura longipedunculata* | 2015090804 | KY884954 | KY884889 | KY884823 | KY884760 |
| *Kadsura longipedunculata* | 2015090805 | KY884955 | KY884890 | KY884824 | KY884761 |
| *Kadsura longipedunculata* | 2015090806 | KY884956 | KY884891 | KY884825 | KY884762 |
| *Kadsura longipedunculata* | 2015090807 | KY884957 | KY884892 | KY884826 | KY884763 |
| *Kadsura longipedunculata* | 2015090808 | KY884958 | KY884893 | KY884827 | - |
| *Kadsura longipedunculata* | 2015090809 | KY884959 | KY884894 | KY884828 | KY884764 |
| *Kadsura longipedunculata* | 2015091001 | KY884765 | KY884829 | KY884895 | KY884960 |
| *Kadsura longipedunculata* | 2015091002 | KY884766 | KY884830 | KY884896 | KY884961 |
| *Kadsura longipedunculata* | 2015091101 | KY884767 | KY884831 | KY884897 | KY884962 |
| *Kadsura longipedunculata* | 2015091102 | KY884963 | KY884898 | KY884832 | KY884768 |
| *Kadsura longipedunculata* | 2015091103 | KY884964 | KY884899 | KY884833 | KY884769 |
| *Kadsura longipedunculata* | 2015091105 | KY884965 | KY884900 | KY884835 | KY884770 |
| *Kadsura longipedunculata* | 2015091801 | KY884966 | KY884901 | KY884836 | KY884771 |
| *Kadsura longipedunculata* | 2015091802 | KY884967 | KY884902 | KY884837 | KY884772 |
| *Kadsura longipedunculata* | 2015092301 | KY884968 | KY884903 | KY884838 | KY884773 |
| *Kadsura longipedunculata* | 2015092302 | KY884969 | KY884904 | KY884839 | KY884774 |
| *Kadsura longipedunculata* | 2015092303 | KY884970 | KY884905 | KY884840 | KY884775 |
| *Kadsura longipedunculata* | 2015092306 | KY884971 | KY884906 | KY884841 | KY884776 |
| *Kadsura longipedunculata* | 2015092307 | KY884972 | KY884907 | KY884842 | KY884777 |
| *Kadsura longipedunculata* | 2015092309 | KY884973 | KY884908 | KY884843 | KY884778 |
| *Kadsura longipedunculata* | 2015092310 | KY884974 | KY884909 | KY884844 | KY884779 |

**Supplementary Table S2**

The cpDNA and ITS haplotypes detected in the three *Kadsura* species of this sdtudy

| Haplotypes of cpDNA | sites | | | | | | | | | | | | | | | | | | | | | | | | | | | | | | | | | | | | | | | | | | | | | | | | | | | |
| --- | --- | --- | --- | --- | --- | --- | --- | --- | --- | --- | --- | --- | --- | --- | --- | --- | --- | --- | --- | --- | --- | --- | --- | --- | --- | --- | --- | --- | --- | --- | --- | --- | --- | --- | --- | --- | --- | --- | --- | --- | --- | --- | --- | --- | --- | --- | --- | --- | --- | --- | --- | --- |
|  | 123 | 135 | 275 | 320 | 348 | 441 | 486 | 666 | 679 | 681 | 684 | 685 | 691 | 692 | 693 | 694 | 695 | 696 | 702 | 703 | 704 | 710 | 711 | 737 | 768 | 769 | 770 | 779 | 783 | 795 | 810 | 818 | 819 | 820 | 824 | 825 | 826 | 827 | 828 | 847 | 920 | 1096 | 1195 | 1308 | 1456 | 1495 | 1530 | 1613 | 1614 | 1615 | 1661 | 1669 |
| C1 | C | T | A | C | G | T | C | A | A | C | T | T | T | T | T | T | C | T | G | G | T | - | A | T | A | A | G | T | C | C | G | - | - | A | - | - | - | - | - | T | - | C | A | G | G | G | T | T | T | C | T | A |
| C2 | C | T | A | C | G | T | C | A | A | C | T | T | T | T | T | T | C | T | G | G | T | - | A | T | A | A | G | T | C | C | T | - | - | A | - | - | - | - | - | T | - | C | A | G | G | G | T | T | T | C | T | A |
| C3 | C | T | A | C | G | T | C | A | A | C | T | T | T | T | T | T | C | T | G | G | T | - | A | T | A | A | G | T | C | C | G | - | - | C | - | - | - | - | - | T | A | C | A | G | G | G | T | T | T | C | T | A |
| C4 | C | T | A | C | G | T | C | A | C | A | C | C | A | G | A | A | A | A | A | A | - | T | - | C | C | T | T | T | C | C | G | - | - | A | - | - | - | - | - | C | - | C | A | G | G | G | T | T | T | C | T | A |
| C5 | C | T | A | C | G | T | C | A | A | C | T | T | T | T | T | T | C | T | G | G | T | - | A | T | A | A | G | T | C | C | G | - | - | C | - | - | - | - | - | T | A | C | A | T | G | G | T | G | A | A | T | A |
| C6 | C | T | A | C | G | T | T | A | A | C | T | T | T | T | T | T | C | T | G | G | T | - | A | T | A | A | G | T | C | C | G | - | - | C | - | - | - | - | - | T | - | C | A | G | G | G | T | T | T | C | T | A |
| C7 | C | T | A | C | G | T | C | G | C | A | C | C | A | G | A | A | A | A | A | A | A | T | A | T | C | T | T | G | T | T | G | T | C | A | T | A | C | T | A | T | A | C | A | G | G | G | T | T | T | C | T | A |
| C8 | C | T | A | C | G | T | C | G | C | A | C | C | A | G | A | A | A | A | A | A | A | T | A | T | C | T | T | G | T | T | G | T | C | A | T | A | C | T | A | T | A | T | G | T | A | A | G | G | A | A | G | G |
| C9 | C | C | A | C | G | C | T | G | C | A | C | C | A | G | A | A | A | A | A | A | A | T | A | T | C | T | T | G | T | T | G | T | C | A | T | A | C | T | A | T | A | T | G | T | A | A | G | G | A | A | G | G |
| C10 | C | C | A | C | G | C | T | G | C | A | C | C | A | G | A | A | A | A | A | A | A | T | A | T | C | T | T | G | T | T | G | T | C | A | T | A | C | T | A | T | - | T | G | T | A | A | G | G | A | A | G | G |
| C11 | T | C | T | C | A | C | T | G | C | A | C | C | A | G | A | A | A | A | A | A | A | T | A | T | C | T | T | G | T | T | G | T | C | A | T | A | C | T | A | T | A | T | G | T | A | A | G | G | A | A | G | G |
| C12 | T | C | T | C | A | C | T | G | C | A | C | C | A | G | A | A | A | A | A | A | A | T | A | T | C | T | T | G | T | T | G | T | C | A | T | A | C | T | A | T | - | T | G | T | A | A | G | G | A | A | G | G |
| C13 | T | C | T | G | A | C | T | G | C | A | C | C | A | G | A | A | A | A | A | A | A | T | A | T | C | T | T | G | T | T | G | T | C | A | T | A | C | T | A | T | - | T | G | T | A | A | G | G | A | A | G | G |
| Haplotypes of ITS | sites | | | | | | | | | | | | | | | | | | | | | | | | | | | | | | | | | | | | | | | | | | | | | | | | | | | |
|  | 19 | 48 | 54 | 61 | 67 | 69 | 71 | 73 | 75 | 86 | 96 | 104 | 116 | 117 | 120 | 126 | 128 | 155 | 177 | 179 | 210 | 214 | 228 | 232 | 248 | 249 | 254 | 256 | 267 | 275 | 279 | 453 | 478 | 482 | 502 | 522 | 529 | 530 | 531 | 537 | 572 | 573 | 608 | 609 | 621 | 624 | 633 | 645 | 651 |  |  |  |
| H1 | C | C | G | C | C | T | C | G | A | C | G | A | A | C | T | C | C | A | C | C | C | - | C | C | G | C | T | G | G | C | C | C | T | C | A | C | C | G | T | C | T | G | G | C | A | T | T | G | C |  |  |  |
| H2 | C | C | G | C | C | T | C | G | A | C | G | A | A | C | T | C | C | A | C | C | C | - | C | C | G | C | T | G | G | C | C | C | T | C | C | C | C | G | T | C | T | G | G | C | A | T | T | G | C |  |  |  |
| H3 | C | C | G | C | C | T | C | G | G | C | G | A | A | C | T | C | C | A | C | C | C | - | C | C | G | C | T | G | G | C | C | C | T | C | A | C | C | G | T | C | T | G | G | C | A | T | T | G | C |  |  |  |
| H4 | C | C | A | T | C | T | C | A | A | T | A | A | A | C | C | C | C | G | C | C | T | A | - | T | A | C | T | C | A | T | T | T | T | C | C | T | C | A | C | T | C | G | A | T | A | C | T | G | T |  |  |  |
| H5 | C | C | A | T | C | T | C | A | A | T | A | A | A | C | T | C | C | G | C | C | T | A | C | T | A | C | T | G | A | T | T | T | T | C | C | T | C | A | T | T | C | A | G | T | A | T | T | G | T |  |  |  |
| H6 | C | C | A | T | C | T | C | A | A | T | A | A | A | C | T | C | C | G | C | C | T | A | C | T | A | C | T | G | A | T | T | T | T | C | C | T | C | A | T | T | C | G | G | T | A | T | T | G | T |  |  |  |
| H7 | C | C | A | T | C | T | C | A | A | T | A | A | A | C | C | C | C | G | C | C | T | A | - | T | A | C | T | C | A | T | T | T | T | C | C | T | C | A | C | T | C | G | G | T | A | C | T | G | T |  |  |  |
| H8 | C | C | A | T | C | T | C | A | A | T | A | A | A | C | C | C | C | G | C | C | T | A | - | T | A | C | T | C | A | T | T | T | T | C | C | T | C | A | C | T | C | G | G | T | A | C | C | G | T |  |  |  |
| H9 | C | C | A | T | C | T | C | A | A | T | A | A | A | C | T | C | C | G | C | C | T | A | - | T | A | C | T | C | A | T | T | T | T | C | C | T | C | A | C | T | C | G | G | T | A | C | T | G | T |  |  |  |
| H10 | C | C | A | T | C | T | C | A | A | T | A | A | A | C | T | C | C | G | C | C | T | A | C | T | A | C | T | G | A | T | T | T | C | C | C | T | C | A | T | T | C | G | G | T | A | T | T | G | T |  |  |  |
| H11 | C | C | A | T | C | T | C | A | A | T | A | A | A | C | T | C | C | G | C | C | T | A | - | T | A | C | T | C | A | T | T | T | T | C | C | T | C | A | C | T | C | G | G | T | A | T | T | G | T |  |  |  |
| H12 | C | C | A | T | C | T | T | A | A | T | A | A | A | C | T | C | C | G | A | C | T | A | - | T | A | C | T | G | A | T | T | T | T | C | C | T | C | A | C | T | C | G | G | T | A | T | T | G | T |  |  |  |
| H13 | C | T | A | T | C | T | C | A | A | T | A | A | A | C | T | C | C | G | C | C | T | A | - | T | A | T | T | G | A | T | T | T | T | C | C | T | C | A | C | T | C | G | G | T | A | T | T | G | T |  |  |  |
| H14 | A | C | A | T | C | T | C | A | A | T | A | A | A | C | T | C | C | G | C | C | T | A | C | T | A | C | T | G | A | T | T | T | T | C | C | T | C | A | T | T | C | G | G | T | A | T | T | G | T |  |  |  |
| H15 | C | C | A | T | C | T | C | A | A | T | G | A | A | C | T | C | C | G | C | C | T | A | - | T | A | C | T | G | A | T | T | T | T | C | C | T | C | A | C | T | C | G | G | T | A | T | T | G | T |  |  |  |
| H16 | A | C | A | T | C | A | C | A | A | T | A | A | A | C | T | C | C | G | C | C | T | A | C | T | A | C | T | G | A | T | T | T | T | C | C | T | C | A | T | T | C | G | G | T | A | T | T | G | T |  |  |  |
| H17 | C | C | A | T | C | T | C | A | A | T | A | A | A | C | T | C | C | G | C | C | T | A | - | T | A | C | T | G | A | T | T | T | T | C | C | T | C | A | C | T | C | G | G | T | A | T | T | G | C |  |  |  |
| H18 | C | C | A | T | A | T | C | A | A | T | G | A | A | C | T | C | C | G | C | C | T | A | - | T | A | C | T | G | A | T | T | T | T | C | C | T | C | A | C | T | C | G | G | T | A | T | T | G | C |  |  |  |
| H19 | C | C | A | T | C | T | C | A | A | T | G | A | A | C | T | C | C | G | C | C | T | A | - | T | A | C | T | G | A | T | T | T | T | C | T | T | C | A | C | T | C | G | G | T | A | T | T | G | T |  |  |  |
| H20 | C | C | A | T | C | T | C | A | A | T | A | A | A | C | T | C | C | G | C | C | T | A | C | T | A | C | T | G | A | T | T | T | T | C | C | T | C | A | C | T | C | G | G | T | A | T | T | G | T |  |  |  |
| H21 | C | C | A | T | C | T | C | A | A | T | A | A | C | C | T | T | T | G | C | C | T | A | C | T | A | C | T | G | A | T | T | T | T | C | C | T | C | A | T | T | C | G | G | T | A | T | T | G | T |  |  |  |
| H22 | C | C | A | T | C | T | C | A | A | T | A | A | A | C | T | T | C | G | C | C | T | A | C | T | A | C | T | G | A | T | T | T | T | C | A | T | C | A | T | T | C | G | G | T | T | T | T | G | T |  |  |  |
| H23 | C | C | A | T | C | T | C | A | A | T | G | A | A | T | T | C | C | G | C | T | T | A | - | T | A | C | T | G | A | T | T | T | T | C | C | T | C | A | C | T | C | G | G | T | A | T | T | G | T |  |  |  |
| H24 | C | T | A | T | C | T | C | A | A | T | A | A | A | C | T | C | C | G | C | C | T | A | - | T | A | C | T | G | A | T | T | T | T | A | C | T | C | A | C | T | C | G | G | T | A | T | T | G | T |  |  |  |
| H25 | C | T | A | T | C | T | C | A | A | T | A | A | A | C | T | C | C | G | C | C | T | A | - | T | A | C | T | G | A | T | T | T | T | C | C | T | C | A | C | T | C | G | G | T | A | T | T | G | T |  |  |  |
| H26 | C | T | A | T | C | T | C | A | A | T | A | A | A | C | T | C | C | G | C | C | T | A | - | T | A | T | T | G | A | T | T | T | T | C | C | T | C | A | C | T | C | G | G | T | A | T | T | G | T |  |  |  |
| H27 | C | T | A | T | C | T | C | A | A | T | A | A | A | C | T | C | C | G | C | C | T | A | - | T | A | C | T | G | A | T | T | T | T | C | C | T | C | A | C | T | C | G | G | T | A | T | T | A | T |  |  |  |
| H28 | C | T | G | C | C | T | C | G | A | C | G | A | A | C | T | C | C | A | C | C | C | - | C | C | G | C | T | G | A | C | C | C | T | C | C | C | T | G | T | C | T | G | G | C | A | T | T | G | C |  |  |  |
| H29 | C | C | A | T | C | T | C | A | A | T | A | G | A | T | T | C | C | G | C | C | T | A | - | T | A | C | C | G | A | T | T | T | T | C | C | T | C | A | C | T | C | G | G | T | A | C | T | G | C |  |  |  |
